# Glucose regulates expression of pro-inflammatory genes, *IL-1β* and *IL-12*, through a mechanism involving hexosamine biosynthesis pathway-dependent regulation of α-E catenin

**DOI:** 10.1042/BSR20211066

**Published:** 2021-06-29

**Authors:** Waruni C. Dissanayake, Jin Kyo Oh, Brie Sorrenson, Peter R. Shepherd

**Affiliations:** 1 Department of Molecular Medicine and Pathology, Faculty of Medical and Health Sciences, The University of Auckland, Private Bag 92019, Auckland 1142, New Zealand; 2 Maurice Wilkins Centre for Molecular Biodiscovery, The University of Auckland, Private Bag 92019, Auckland 1142, New Zealand

**Keywords:** alpha catenin, hexosamine biosynthesis pathway, inflammation, macrophages

## Abstract

High glucose levels are associated with changes in macrophage polarisation and evidence indicates that the sustained or even short-term high glucose levels modulate inflammatory responses in macrophages. However, the mechanism by which macrophages can sense the changes in glucose levels are not clearly understood. We find that high glucose levels rapidly increase the α-E catenin protein level in RAW264.7 macrophages. We also find an attenuation of glucose-induced increase in α-E catenin when hexosamine biosynthesis (HB) pathway is inhibited either with glutamine depletion or with the drugs azaserine and tunicamycin. This indicates the involvement of HB pathway in this process. Then, we investigated the potential role of α-E catenin in glucose-induced macrophage polarisation. We find that the reduction in α-E catenin level using siRNA attenuates the glucose-induced changes of both IL-1β and IL-12 mRNA levels under LPS-stimulated condition but does not affect TNF-α expression. Together this indicates that α-E catenin can sense the changes in glucose levels in macrophages via HB pathway and also can modulate the glucose-induced gene expression of inflammatory markers such as *IL-1β* and *IL-12*. This identifies a new part of the mechanism by which macrophages are able to respond to changes in glucose levels.

## Introduction

Macrophages are highly sensitive to environmental stimuli. To acquire distinct functional phenotype against host cell infection, macrophages can be polarised into either pro-inflammatory cytokine secreting-classically activated (M1) or anti-inflammatory cytokine secreting-alternatively activated (M2) phenotype. A sustained or even short-term exposure to high glucose levels is known to induce M1 macrophage polarisation [1,2]. This has the potential to be relevant in disease such where derangements in glucoregulatory mechanisms play an important role. One such disease is COVID-19 as elevated glucose levels favour SARS-CoV2 infection and effects of glucose levels on macrophage function have been shown to play an important role in this [3,4]. Another example is Type 2 diabetes where hyperglycaemia leads to the accumulation of macrophages and other innate immune cells with M1 phenotype [5]. Moreover, it has been shown that M1 macrophages highly depend on glycolysis as the energy source [6,7], while M2 macrophages rely on fatty acid oxidation [8,9]. These studies suggest that glucose metabolism is involved in determining the phenotype of macrophages and thereby regulating inflammatory responses. However, the underlying mechanism is not fully understood.

There are several pathways involved in glucose-sensing mechanisms such as ATP-sensitive K^+^ channels [10], flux through the hexosamine biosynthesis (HB) pathway [11], AMP-activated protein kinase [12] and activated PKC [13]. Previously, we have shown that exposure of macrophage cell lines RAW264.7 and J774.2 to high levels of glucose increases the β-catenin protein level via the HB pathway [14]. β-catenin is a major component of adherens junction, which connects cadherin to α-catenin protein. Wnt/β-catenin pathway is known to promote M2 macrophage polarisation [15] and IL4-induced multinucleated giant cell formation [16]. β-catenin also stabilises α-catenin by preventing the proteasomal degradation [17]. Our previous studies found that glucose stimulation increases the α-E catenin level in rat pancreatic β-cell models (INS-1E and INS-832/3) [18].

In the current study, we sought to determine whether changes in glucose levels regulate α-E catenin levels in RAW 264.7 macrophages and to assess impacts this could have. We found that changes in glucose levels in RAW 264.7 rapidly alter the level of α-E catenin via a mechanism that requires activity of the HB pathway. We show changes in α-E catenin are associated with changes in expression of pro-inflammatory marker genes. Taken together, these data suggest that α-E catenin protein is regulated by the HB pathway and this contributes to changes in M1/M2 macrophage polarisation mediated by changes in glucose levels.

## Materials and methods

### Cell culture

RAW264.7 cells were maintained in RPMI 1640 medium supplemented with 10% (v/v) newborn calf serum, 100 units/ml penicillin and 100 μg/ml streptomycin (all from Life Technologies). Cells were cultured on 12-well format and used for experiment when they were >90% confluent. Cells were serum starved overnight in RPMI medium in the presence of 0.5 mM glucose and treated with glucose, glucosamine or other inhibitors as indicated. All the reagents were purchased from Sigma–Aldrich.

### Cell lysate preparation

After treatments, cells were rinsed twice with 0.5 ml of ice-cold 1× PBS and lysates were collected in buffer containing 20 mM Tris/HCl (pH 7.5), 150 mM NaCl, 1 mM EDTA, 1 mM EGTA, 1% Triton X-100, 2.5 mM sodium pyrophosphate, 1 mM β-glycerol phosphate, 1 mM vanadate, 100 mM NaF, 1 mM 4-(2-aminoethyl) benzenesulfonyl fluoride hydrochloride (AESBF), 4 µg/ml leupeptin, and 30 µM N-[N-(N-Acetyl-l-leucyl)-l-leucyl]-l-norleucine (ALLN), 4 µg/ml aprotinin, 0.4 µg/ml pepstatin. Cell lysates were centrifuged at 16100×***g*** for 10 min, and supernatants were subjected to polyacrylamide gel electrophoresis for Western blotting.

### Western blot analysis

After protein transfer, nitrocellulose membranes were incubated with primary antibodies against α-E catenin (1:1000; Cell Signaling Technology, catalogue number #240) and α-tubulin (1:20,000; Sigma–Aldrich, catalogue number T6074) at 4°C. After overnight incubation with primary antibodies, membranes were washed and incubated with respective secondary antibodies anti-rabbit IgG HRP (1:10000 Santa Cruz Biotechnology) or anti-mouse IgG HRP (1:20,000; Sigma–Aldrich) for 1 h at room temperature and developed with Clarity™ Western ECL substrate (Bio-Rad Laboratories).

### siRNA transfection

α-E catenin siRNA was transfected to RAW 264.7 macrophages using Nepagene electroporator as per instruction manual. Briefly, 250 nM of siRNA was mixed with 1 × 10^7^ cells/100 μl of RAW 264.7 cells in Opti-Mem and electroporated using below conditions. Pouring pulse conditions are voltage at 175 V, pulse length 5 ms, pulse interval 50 ms, 2 pulses, 10% decay rate and + polarity. Transfer pulse conditions are 20 V voltage, 20 ms pulse length, 50 ms pulse interval, 5 pulse, 40% decay rate. Cells were used for experiments 48–72 h after transfection.

### LPS stimulation

Seventy-two hours after siRNA transfection, RAW 264.7 cells were treated with 100 ng/ml LPS for 8 h in the presence of either 0.5 or 20 mM glucose in serum-free media. After treatments, cells were washed with 1× PBS and used for RNA isolation.

### Real-time PCR

RNeasy Mini Kit (Qiagen) was used to extract total RNA from cells. Samples were then treated with DNase I (Life Technologies) to eliminate genomic DNA. cDNA synthesis (reverse transcription) was performed by using the qScript cDNA Super Mix kit (Dnature) with the same amount of RNA added for all samples. The cDNA synthesis conditions were as follows: 5 min at 25°C > 30 min at 42°C > 5 min at 85°C > hold at 4°C. cDNA samples were loaded on to a 394-well PCR plate prior to real-time PCR. In addition, DNase-free water was included as a non-template control. Each reaction was consisted of 5 μl qScript cDNA Super Mix, 5 ng cDNA (equivalent to RNA added) and 400 nM primers and water (up to 10 μl). PowerUp SYBR Green Master Mix (Applied Biosystems) was used for qPCR. The qPCR conditions were as follows: 2 min at 50°C > 10 min at 95°C > 40 cycles (15 s at 95°C, 1 min at 60°C) > 15 s at 95°C > 1 min at 60°C > 15 s at 95°C. The double delta *C*_t_ analysis was performed to calculate the gene expression fold change in relation to the control. A single distinct peak in melt curve was checked to validate the qPCR in each well.

### Statistical analysis

Results are presented as means ± S.E.M. with the number of experiments indicated in the legend. Statistical significance was assessed using Student’s *t* test or two-way ANOVA as indicated in figure legends. Statistical significance is displayed as *P<0.05 or **P<0.01. Statistical analyses were performed using statistical software package GraphPad Prism 6.0 (GraphPad Software Inc.).

## Results

**Figure 1:**
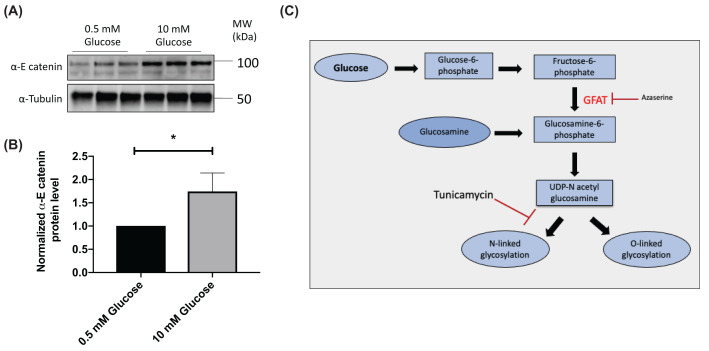
High glucose level increases the α-E catenin level in RAW 264.7 cells After overnight serum starvation in RPMI medium with 0.5 mM glucose, RAW 264.7 cells were treated with either 0.5 or 10 mM glucose for 2 h. (**A**) Cell lysates were subjected to the Western blot analysis using α-E catenin and α-tubulin antibodies. (**B**) Densitometry analysis showing protein expression of α-E catenin normalised to α-Tubulin. Data represent mean ± S.E.M of three independent experiments. (**C**) Schematic diagram of hexosamine biosynthesis pathway. The unpaired *t* test was used to assess statistical significance, *P<0.05.

**Figure 2:**
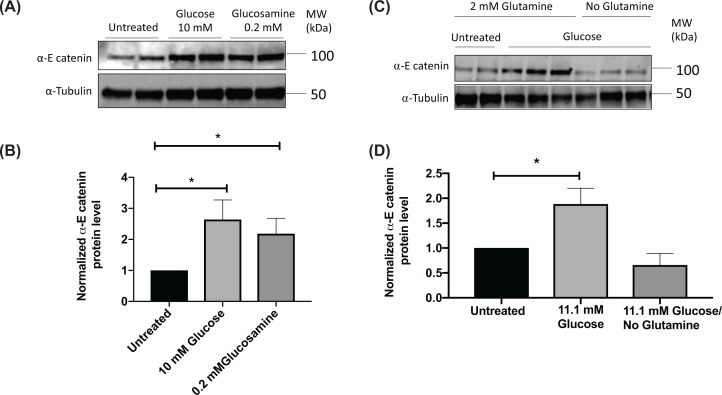
Glucosamine increases the α-E catenin level in RAW 264.7 cells and glutamine is required for glucose-induced increase in α-E catenin RAW 264.7 cells were treated with indicated concentrations of glucose or glucosamine for 2 h after overnight serum starvation in the presence of 0.5 mM glucose. (**A**) Cell lysates were subjected to the Western blot analysis using α-E catenin and α-Tubulin antibodies. (**B**) Densitometry analysis of α-E catenin protein expression in Western blot image relative to the α-Tubulin. RAW 264.7 cells were treated with indicated concentrations of glucose in the presence or absence of glutamine for 2 h. (**C**) Cell lysates were subjected to the Western blot analysis using α-E catenin and α-Tubulin antibodies. (**D**) Densitometry analysis showing protein expression of α-E catenin normalised to α-Tubulin. Data represent mean ± S.E.M of at least four independent experiments. The unpaired *t* test was used to assess statistical significance, *P<0.05.

**Figure 3:**
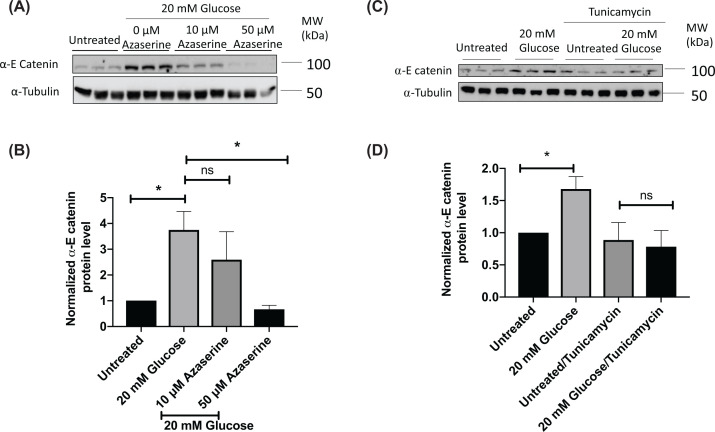
Azaserine and tunicamycin attenuate glucose-induced increase in α-E catenin After overnight serum starvation in RPMI medium with 0.5 mM glucose, RAW 264.7 cells were treated with 20 mM glucose in the presence or absence of azaserine for 4 h. (**A**) Cell lysates were subjected to the Western blot analysis using α-E catenin and α-Tubulin antibodies. (**B**) Densitometry analysis showing protein expression of α-E catenin normalised to α-Tubulin. Data represent mean ± S.E.M of three independent experiments. The unpaired *t* test was used to assess statistical significance, *P<0.05. RAW 264.7 cells were treated with 20 mM glucose in the presence or absence of tunicamycin (10 μg/ml) for 2 h. (**C**) Cell lysates were subjected to the Western blot analysis using α-E catenin and α-Tubulin antibodies. (**D**) Densitometry analysis showing protein expression of α-E catenin normalised to α-Tubulin. Data represent mean ± S.E.M of four independent experiments. The unpaired *t* test was used to assess statistical significance, *P<0.05.

We found that high levels of glucose cause an increase in α-E catenin protein levels in the RAW 264.7 macrophage model (Figure 1A,B), consistent with our previous findings in rat pancreatic β-cells [18]. As we have previously found that β-catenin levels increased with glucose stimulation in macrophage cell lines through the HB pathway [14], we speculated that the same pathway might account for the glucose-induced up-regulation of α-E catenin. To investigate this possibility, RAW 264.7 cells were treated with glucosamine. Glucosamine is converted into glucosamine-6-phosphate (GlucN-6-P), bypassing the first three steps required for glucose in the HB pathway (Figure 1C). Glucosamine increased α-E catenin levels in macrophages in a similar manner to that seen with high glucose (Figure 2A,B). The conversion of fructose-6-phosphate into GlucN-6-P (the rate limiting-step of the HB pathway) is regulated by glutamine:fructose-6-phosphate-amidotransferase (GFAT) enzyme, which requires glutamine as a co-substrate [19]. Therefore, to further confirm that the HB pathway is responsible for modulating the level of α-E catenin, we treated RAW 264.7 cells with high glucose in the absence of glutamine and observed no significant change in α-E catenin level (Figure 2C,D). In line with this finding, when GFAT enzyme was inhibited with azaserine, the glucose effect on α-E catenin levels were attenuated (Figure 3A,B). The HB pathway mediates the glycosylation of proteins, which is important for protein stability, protein activity, cell–cell communication and signal transduction [20,21]. Next, we treated cells with tunicamycin, which is an inhibitor of N-linked glycosylation. The glucose effect on α-E catenin was attenuated in the presence of tunicamycin (Figure 3C,D), indicating that the regulation of α-E catenin level relies on N-linked glycosylation. These findings suggest that α-E catenin level in RAW264.7 is regulated via the HB pathway involving N-linked glycosylation.

**Figure 4:**
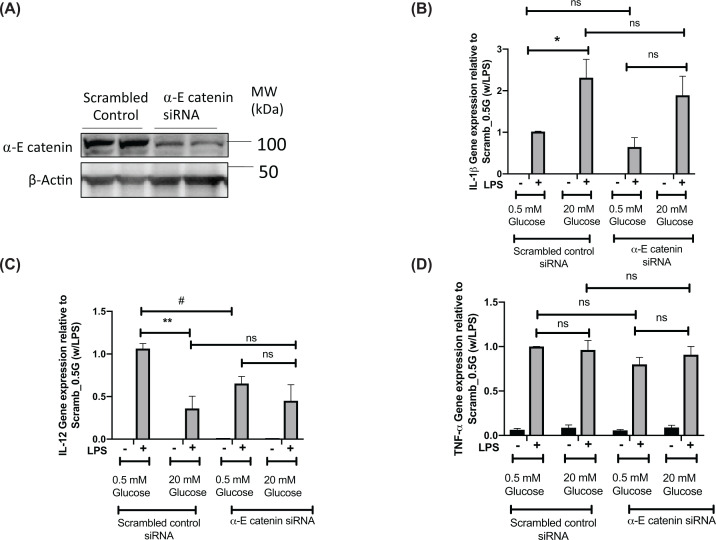
α-E catenin knockdown blunts glucose-induced increase in *IL-1β* and glucose-induced decrease in *IL-12* Forty-eight hours after siRNA transfection, RAW264.7 cells were either untreated or treated with LPS in the presence of 0.5 or 20 mM glucose for 8 h. (**A**) Cell lysates were subjected to the Western blot analysis using α-E catenin and α-Tubulin antibodies. (**B–D**) qRT-PCR showing relative gene expression of *IL-1β*, *IL-12* and TNF-α. Data represent mean ± S.E.M of three independent experiments, assessed by two-way Anova *P<0.05, **P<0.01 or *t* -test, ^#^*P*<0.05.

We next investigated the role of α-E catenin in the function of the RAW cells using siRNA to reduce α-E catenin. We electroporated RAW 264.7 cells with an α-E catenin siRNA, which led to at least 50% reduction in levels of α-E catenin (Figure 4A). We then treated cells with either low (0.5 mM glucose) or high glucose (20 mM glucose) in the presence or absence of LPS. We found that decreased expression of *Ctnna1* blunts glucose-induced up-regulation of *IL-1β* under the LPS-stimulated condition (Figure 4B). A more profound effect was observed on *IL-12* expression where we observed the decreased expression in the α-E catenin knockdown samples and a loss of the glucose-mediated reduction in LPS-induced *IL-12* expression (Figure 4C). Under LPS-stimulated conditions, we did not observe any effect of α-E catenin knockdown on *TNF-α* at either low or high glucose conditions (Figure 4D).

## Discussion

Macrophages have phenotypic plasticity which is an important for their function in inflammation. Polarisation towards the M1 state can be induced by the TLR agonist LPS [22] and here we see this increase levels of M1 polarisation markers, namely *IL-1β* and *IL-12* and *TNF-α*. High glucose levels favour macrophage polarisation towards the M1 phenotype, characterised by the secretion of pro-inflammatory cytokines which induces inflammation of surrounding environment. So we were interested in how changes in α-catenin could interact with glucoses effects on this. Therefore, most novel finding of the current study is identification of a previously unreported role of α-E catenin in glucose-mediated effects on macrophage polarisation. This shown by the impacts of reducing α-E-catenin on glucose modulatory effects on pro-inflammatory genes *IL-1β* and *IL-12*. This implicates α-E catenin as a new component of mechanisms involved in switching macrophages between M1 and M2 states. We find that that α-E catenin protein level is increased upon glucose stimulation using the RAW 264.7 cell line as a macrophage cell model via the HB pathway which could thus contribute to the changes in macrophage function known to be brought on by changes in glucose levels [1,2] .

One outcome of the HB pathway is protein glycosylation and a lack of proper protein glycosylation results in accumulation of misfolded proteins in the endoplasmic reticulum (ER) which results in ER stress [23]. Thus, the finding that the glycosylation inhibitor tunicamycin blocks glucose-induced increases in α-E catenin in our study suggests normal ER function would be required for this mechanism of α-E catenin regulation. It is notable that a separate proteomic approach has also shown that ER stress also reduces both α-catenin and β-catenin protein levels in HeLa cells and that ER stress was reducing catenin levels by increasing rates of proteasomal degradation [23]. Proteasomal turnover of α and β-catenin is associated with increased CK1 and GSK3-mediated phosphorylation of β-catenin at Ser^37^/Thr^41^ [24,25]. Tunicamycin regulates GSK3 in the proteomics study [23] and studies in other cell types also show that tunicamycin activates GSK3 which would be consistent with reductions in α and β-catenin [26–29]. However, we do not see any changes on GSK3β levels or phosphorylation status despite seeing a reduction in β-catenin Ser^37^/Thr^41^ phosphorylation [14]. CK1 activity has also been reported to a change in response to changes in glucose levels in some cell types [30] and tunicamycin may increase CK1 activity [31], although it is not known if this occurs in macrophages. Therefore, the mechanisms linking changes in glucose-induced glycosylation to changes in α-E catenin levels remain to be elucidated.

In a similar study, it has been shown that high glucose levels decrease the mRNA and protein levels of silent mating type information regulation 2 homologue 1 (SIRT1) and the modulation of SIRT1 by inhibitors or siRNA further increases the glucose-induced increase in pro-inflammatory markers IL-1β and TNF-α [1]. A similar effect of glucose on pro-inflammatory cytokines to what we have seen in RAW264.7 cell line has been observed in BMDM only after long-term (7 days) high glucose treatment [2]. Here they have shown that long-term high glucose treatment increases IL-1β, but decreases IL-12 expression, however short-term glucose treatment only affects IL-1β expression [2]. These glucose-induced changes in IL-1β could have impacts on metabolism as elevation in IL-1β have been shown to have detrimental impacts on regulation of metabolism [32]. These impacts on metabolism may be part of a physiologically relevant cycle involved in regulating glucose metabolism as acute increases in circulating levels of IL-1β have been observed postprandially [32,33]. Of relevance to our work is the evidence that these changes are due to glucose-induced production of IL-1β in macrophages in the gut.

In addition, our findings could also have implications for pathology associated with Type 2 diabetes as in our previous work, we also have demonstrated that α-catenin inhibits insulin secretion in pancreatic β-cells [18]. Type 2 diabetes is characterised by β-cell failure [34] in which chronic inflammation involving macrophages has been implicated [35,36]. Thus, it will be worth investigating how glucose-induced changes in α-E catenin in both β-cells and macrophages might combine in promoting progression of this disease.

## Data Availability

No large datasets are associated with the present study. All raw data for Western blots and qPCR are available upon request.

## Open Access

Open access for this article was enabled by the participation of University Of Auckland in an all-inclusive *Read & Publish* pilot with Portland Press and the Biochemical Society under a transformative agreement with CAUL.

## Abbreviations

BMDM: bone marrow derived macrophages
CK1: casein kinase 1
ER: endoplasmic reticulum
GFAT: glutamine:fructose-6-phosphate-amidotransferase
GlucN-6-P: glucosamine-6-phosphate
GSK3: glycogen synthase kinase 3
HB: hexosamine biosynthesis
IL: Interlukin
LPS: lipopolysaccharides
PKC: protein Kinase C
